# Clinicopathological observation of adult malignant ectodermal mesenchymoma: A case report and literature review

**DOI:** 10.1097/MD.0000000000045908

**Published:** 2025-12-26

**Authors:** Le Xie, Yingxin Huang, Rongjun Mao

**Affiliations:** a Department of Pathology, The Eighth Clinical Medical College of Guangzhou University of Chinese Medicine, Foshan, Guangdong, China; b Department of Pathology, Foshan Hospital of Traditional Chinese Medicine, Foshan, Guangdong, China.

**Keywords:** clinicopathology, malignant ectomesenchymoma, prognosis, treatment

## Abstract

**Rationale:**

Malignant ectomesenchymoma (MEM) is a relatively rare soft tissue tumor, which predominantly occurs in infants or children under 15 years of age. The classic sites of occurrence are the pelvic perineal region and genitourinary system. Compared to pediatric cases, adult cases exhibit certain differences in clinicopathological features and are more prone to misdiagnosis. A thorough comprehension of their clinicopathologic features is crucial for precise diagnosis and effective management.

**Patient concerns:**

A 23-year-old male had a lesion located on the right side of his head and face, and the disease course lasted 13 months. A retrospective analysis was conducted on the clinicopathological data, histopathological features, immunophenotype, and molecular pathological changes.

**Diagnoses:**

The tumor was composed of embryonal rhabdomyosarcoma and undifferentiated neuroectodermal components. Immunohistochemistry showed positivity for rhabdomyosarcoma markers such as Desmin, MyoD1, and Myogenin in the rhabdomyosarcomatous component and positivity for neuroectodermal markers, including synaptophysin and chromogranin A in the neuroectodermal component. No specific molecular alterations were detected in genetic testing.

**Interventions:**

This is a case of initial misdiagnosis, multimodal comprehensive treatment combining surgery with chemotherapy was the primary therapeutic approach, However, poor clinical outcomes were observed.

**Outcomes:**

Despite aggressive treatments, the patient died of the disease after 1 year of follow-up.

**Lessons:**

Adult MEM is a biphenotypic sarcoma composed of rhabdomyosarcoma and neuroectodermal tissues. Morphological and immunohistochemical interpretation is crucial for diagnosis. treatment approaches and prognosis are correlated with the completeness of tumor resection and the differentiation degree of both components.

## 1. Introduction

Malignant ectomesenchymoma (MEM) is a relatively rare soft tissue tumor composed of rhabdomyosarcoma (RMS) and neuroectodermal or neuronal components. The 2020 World Health Organization classification categorizes MEM as a variant of RMS.^[1]^ Currently, fewer than 150 cases have been reported, with most cases occurring in the genitourinary region of infants and young children. As well as being extremely rare, adult cases of MEM are prone to be missed or misdiagnosed during initial diagnosis due to their nonspecific clinical and imaging manifestations as well as their variable microscopic morphology. In this article, we report a case of adult temporal MEM diagnosed through consultation at our hospital, and we analyze its clinicopathological characteristics in conjunction with a literature review. The aim of this study was to enhance the understanding of this rare tumor, further accumulate cases to expand the tumor spectrum, and reduce misdiagnosis. This report represents the first international summary study on adult MEM.

## 2. Materials and methods

### 2.1. Source of materials

One case of adult MEM was extracted from the Difficult Pathology Consultation Database of Foshan Hospital of Traditional Chinese Medicine, Foshan, China. Clinical, imaging, and follow-up data of this case were collected, and pathological sections were reexamined for confirmation.

### 2.2. Methods

The white tissue sections were subjected to hematoxylin and eosin staining. Immunohistochemistry was performed using the SuperVision method, with all antibodies, including vimentin, AE1/AE3, S-100, SOX10, synaptophysin (Syn), chromogranin A (CgA), Desmin, Myogeinin, MyoD1, CD34, SMA, and Ki67, purchased from Shanghai Jiehao Biotechnology Co., Ltd, Shanghai, China.

## 3. Results

### 3.1. Clinical data

A 23-year-old male had a lesion located on the right side of his head and face, and the disease course lasted 13 months. The treatment timeline is shown in Table 1. The patient had no family history or history of severe trauma, and laboratory tests showed no abnormalities.

**Table 1:** The treatment timeline of our case.

Timeline	Event	Treatment	Outcome
Initial discovery	Noticed a small nodule in the frontotemporal region; grew rapidly from 3–9 cm within 2 mo	N	N
First treatment	Underwent mass resection at a local hospital; pathology confirmed embryonal rhabdomyosarcoma (ERMS)	Mass resection	Diagnosed with ERMS
Post-op chemo	Initiated chemotherapy after surgery	VDC + i.e. regimen (vincristine, doxorubicin, cyclophosphamide + ifosfamide, etoposide)	N
First recurrence	Tumor recurred during the 4th cycle of chemotherapy	N	Tumor recurrence
Second surgery	Underwent wide local excision + partial parotidectomy + right level II lymph node dissection	Extensive resection	N
Second recurrence	Ipsilateral cheek metastasis detected about 1 mo after the second surgery	N	Cheek metastasis
Continued chemo	Continued adjuvant VDC + i.e. chemotherapy	VDC + i.e. regimen	Metastatic lesion reduced in size
Third recurrence	Ipsilateral post-auricular mass appeared during the 11th cycle of chemo (~4 mo post-op); biopsy confirmed RMS metastasis	N	Post-auricular metastasis
Regimen change	Switched to VI regimen (vincristine + irinotecan) with an increased dose of Irinotecan	VI regimen	Mass continued to grow after 4 cycles, with rapid spread
Final action	Referred to our hospital for consultation	N	N

### 3.2. Physical examination

**Figure 1.**
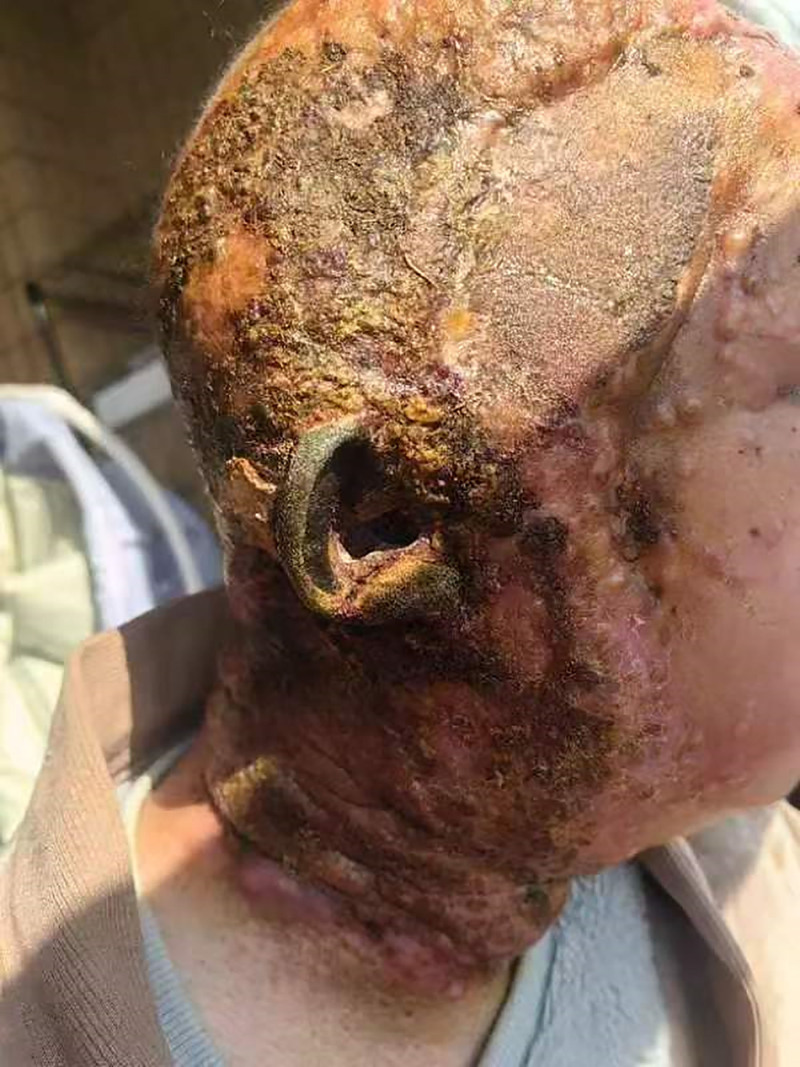
The left forehead, temporal region, parotid area, cheek, and postauricular region extending to the neck exhibit diffuse nodular swelling.

The left forehead, temporal region, parotid area, cheek, and postauricular region extending to the neck exhibit diffuse nodular swelling, with the lesion measuring approximately 30 cm × 19 cm in size (Fig. 1). Physical examination also revealed the following: swelling of the ipsilateral orbit and lips, difficulty in opening the eyes, mouth opening limited to 2.7 cm, no redness or swelling observed in the ipsilateral parotid duct, and no purulent discharge present.

### 3.3. Pathological examination

#### 3.3.1. Microscopic morphology

**Figure 2.**
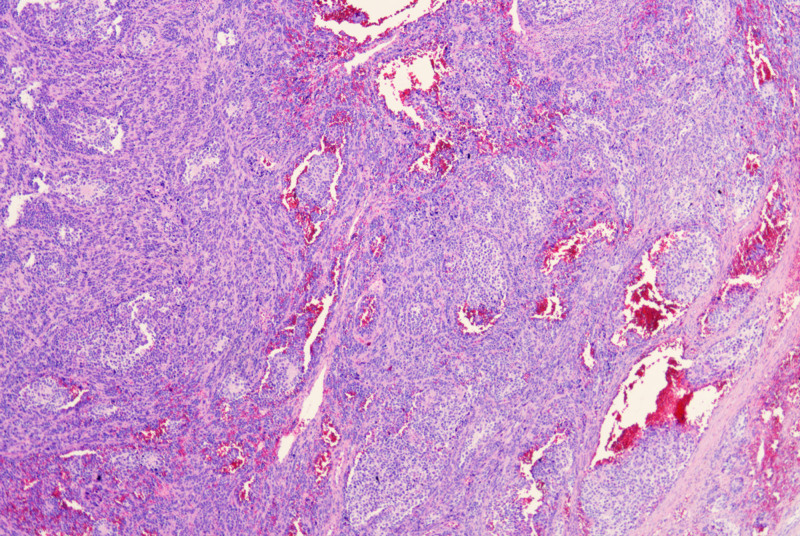
Evident intravascular tumor emboli were observed (HE, ×40). HE= hematoxylin and eosin.

**Figure 3.**
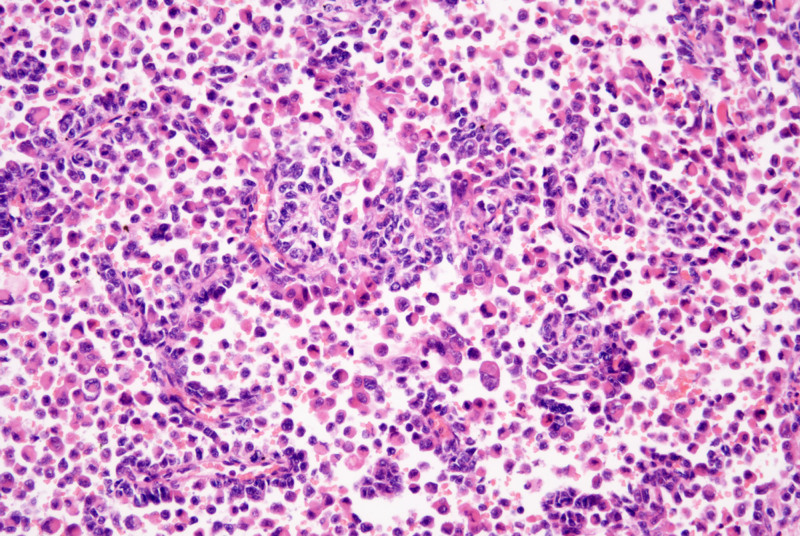
Small cell nests are distributed in a background of scattered lymphocytes and epithelioid cells (HE, ×200). HE = hematoxylin and eosin.

**Figure 4.**
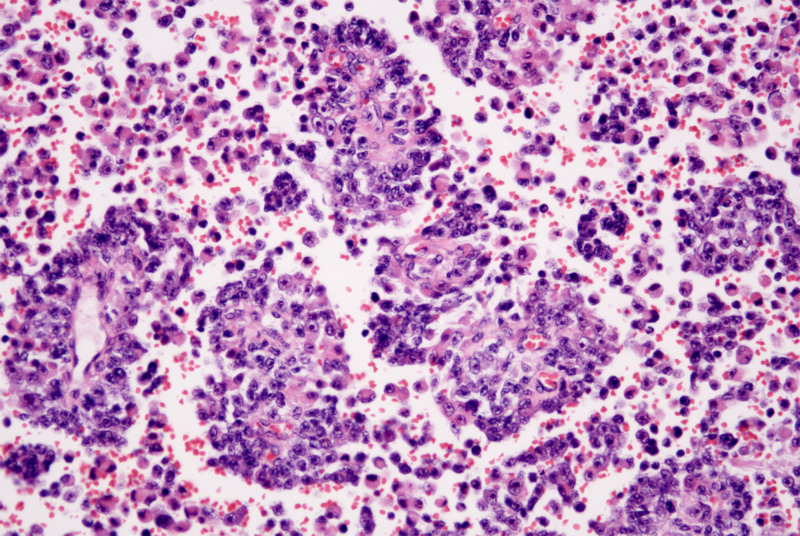
Small round cells arranged in nested, rosette-like, and pseudopapillary patterns (HE, ×200). HE = hematoxylin and eosin.

**Figure 5.**
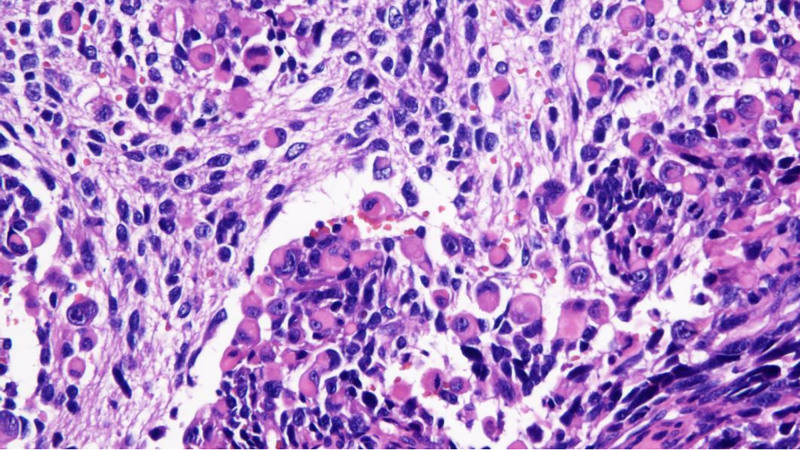
Under high magnification, eosinophilic tumor cells exhibited myocyte-like morphology (HE, ×400). HE= hematoxylin and eosin.

The tumor was composed of eosinophilic epithelioid cells and small round cells with bare nuclei in varying proportions and configurations. Under low magnification, the tumor tissue exhibited indistinct borders and extended pseudopod-like projections into the surrounding skeletal muscle and subcutaneous adipose tissue. Evident intravascular tumor emboli were observed (Fig. 2), with the tumor exhibiting a biphasic architecture. Small round cells arranged in nested, rosette-like, and pseudopapillary patterns were intermingled against a background of diffusely distributed lymphoid cells and eosinophilic epithelioid cells (Figs. 3 and 4). The former accounts for approximately 75%, while the latter accounts for approximately 25%. The interstitial fibers were sparse, with abundant thin-walled small blood vessels, showing signs of erythrocyte extravasation and focal hemorrhage and necrosis. Under high magnification, eosinophilic tumor cells exhibited myocyte-like morphology (Fig. 5), appearing as plump spindle-shaped, polygonal, or roundish cells of varying sizes. The nuclei were mostly eccentrically located and hyperchromatic, showing certain atypia manifested as irregular nuclear contours, hyperchromasia, and occasional bizarre nuclear morphology, with observable pathological mitotic figures. Clustered small round cells with scanty cytoplasm, uneven nuclear membrane thickness, and prominent nucleoli were present. Tumor mitotic figures are readily observed, with visible hemorrhage and necrosis. Tumor metastasis is identified in multiple lymph nodes resected during surgery.

#### 3.3.2. Immunophenotype

**Figure 6.**
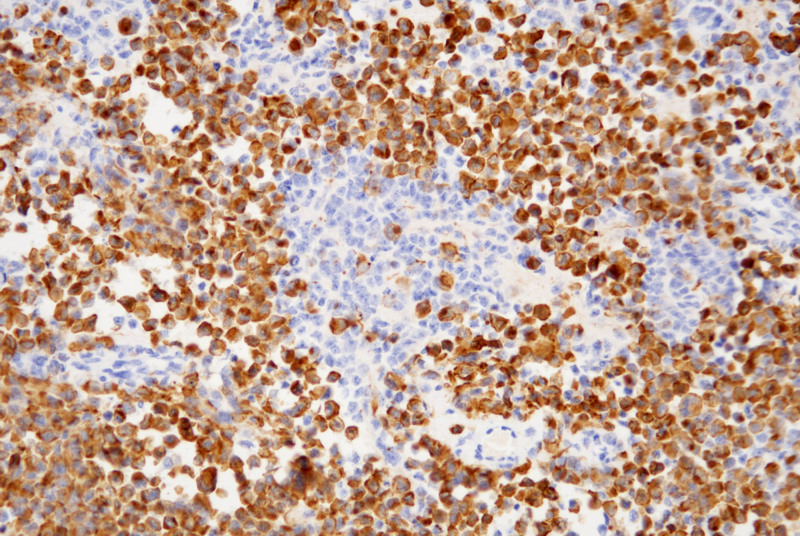
Lymphoid cells and eosinophilic epithelioid cells mainly express Desmin, the positive staining is localized in the cytoplasm (IHC, ×200). IHC = immunohistochemistry.

**Figure 7.**
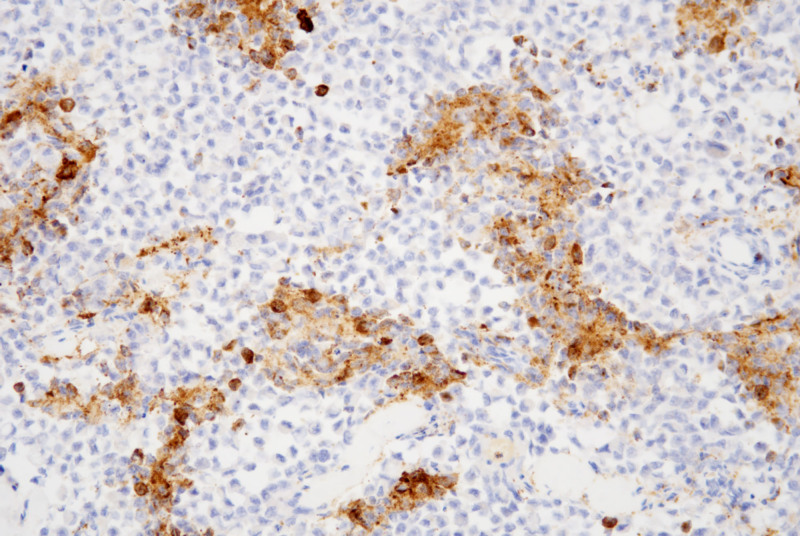
The nested, rosette-like, and pseudopapillary configurations of small round cells predominantly express Syn, the positive staining is localized in the cytoplasm (IHC, ×200). IHC = immunohistochemistry, Syn = synaptophysin.

**Figure 8.**
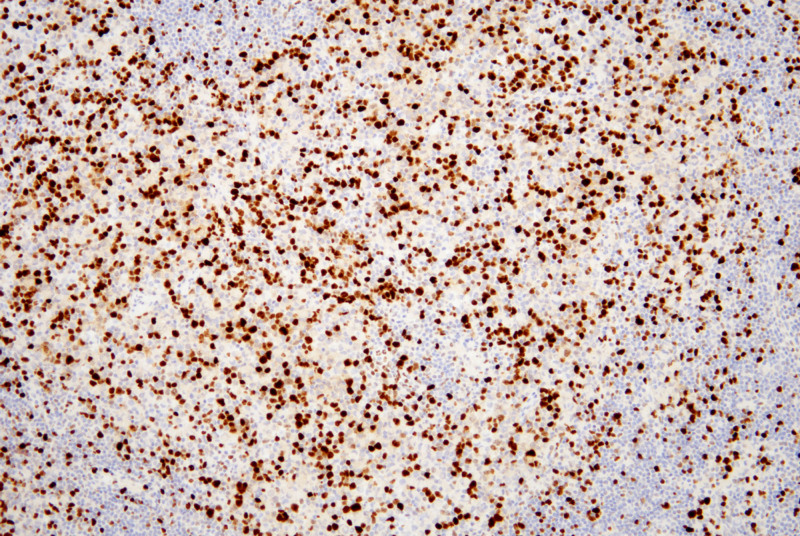
Ki67 positivity of approximately 50–70%, the positive staining is localized in nucleus (IHC, ×40). IHC = immunohistochemistry.

Lymphoid cells and eosinophilic epithelioid cells mainly express Desmin (Fig. 6), MyoD1, and Myogenin, while the nested, rosette-like, and pseudopapillary configurations of small round cells predominantly express Syn (Fig. 7) and CgA, with Ki67 positivity of approximately 50 to 70% (Fig. 8).

#### 3.3.3. Molecular test results

The fluorescence in situ hybridization test at our hospital showed an NCOA2 break (–); the external hospital reverse transcription-polymerase chain reaction test: MyoD1 mutation (–). The Next Generation Sequencing test conducted at an external hospital revealed a *NOTCH2* frameshift mutation (significance unclear).

#### 3.3.4. Prognosis and follow-up

The final diagnosis was MEM, and the patient died of the disease after 1 year of follow-up.

## 4. Discussion

MEM was first summarized and named by Naka et al^[2]^ in 1975, initially defined as a malignant tumor composed of 2 components derived from mesodermal mesenchymal elements and neuroectoderm. The mesenchymal components include RMS, liposarcoma, and chondrosarcoma, among others. Subsequently, with in-depth research on dedifferentiated liposarcoma, mesenchymal chondrosarcoma, and other related soft tissue tumors, the definition of MEM gradually narrowed. The fifth edition (2020) of the World Health Organization Classification of Soft Tissue Tumors classified MEM as a subtype of RMS. Defined as an RMS containing neuronal or neuroblastic components, this tumor is relatively rare and predominantly occurs in infants or children under 15 years of age. The classic sites of occurrence are the pelvic perineal region and genitourinary system. Compared to pediatric cases, adult cases exhibit certain differences in clinicopathological features and are more prone to misdiagnosis.

### 4.1. Clinical characteristics

A search and statistical analysis of 10 previously reported cases^[3–12]^ and the current consultation case (Table 2) revealed that the median age of onset for MEM was 46 years, with more than half of the patients being in the 30 to 50-year age group. Males predominated, with a male-to-female ratio of 8:3. With the exception of 3 cases located in the urogenital system (renal pelvis, testis, and uterus), the masses were almost exclusively found in the head and neck region (8 cases), particularly in the nasal cavity and paranasal sinuses (5 cases), followed by the soft tissues of the frontal-temporal region and the meninges. The tumor manifests as a localized space-occupying lesion or exophytic mass. When located in the nasal cavity, it mostly presents as a polypoid with well-defined margins in the early stages,^[5]^ is painless, and shows no abnormalities in tumor markers.^[10]^ Larger tumors may cause obstruction and compression symptoms in the corresponding areas,^[4–8,11]^ including hematuria, headache, nasal congestion, rhinorrhea, and epistaxis. Cases originating in the ethmoid sinus^[6]^ presented with eye distension and proptosis as the initial symptoms due to tumor invasion of the orbit and compression of the medial rectus muscle, while the case located in the left frontotemporal parietal lobe^[11]^ manifested as weakness of the right limb caused by tumor edema compressing the corresponding functional area. The tumor may rapidly increase in size within a short period, as seen in our hospital case, while scrotal case developed multiple pulmonary metastases within 2 weeks after radical orchiectomy.^[10]^ Gross examination of the resected tumor shows no specific features, mostly presenting with a firm texture and grayish-white cut surface. No special associated past medical history or predisposing factors were observed in the case groups.

**Table 2:** **Clinical and pathological data of 11 adult MEM cases**.

Case	Literature	Age (yr)	Sex	Site of onset	Histo-pathology	Molecular testing	Treatment method	Follow-up
1	Paulus W (1991) ^[3]^	18	M	Temporal bone and meninges	ERMS + GC	N	Unknown	Unknown
2	Chu PG (2002) ^[4]^	68	M	Renal pelvis + regional lymph nodes	ARMS + GN	N	Surgery + chemotherapy	No recurrence or metastasis during the 6-year follow-up period
3	Brehmer D (2003) ^[5]^	62	M	Nasal cavity	PRMS + UNB	N	Surgery + radiotherapy	No recurrence or metastasis during the 36-mo follow-up period
4	Cergnul M (2007) ^[6]^	62	M	Nasal cavity, ethmoid sinus	ERMS + GNB	N	Surgery + chemoradiotherapy	No recurrence or metastasis was observed during the 18-mo follow-up period
5	Vinck A-S (2011) ^[7]^	36	F	Right sphenoid sinus	ERMS + PDNB	N	Chemotherapy + radiotherapy	Significant decrease in tumor volume, achieving tumor-bearing survival for 28 mo post-treatment
6	Patil CN (2011) ^[8]^	43	F	Left nasal cavity, maxillary sinus, frontal sinus + left cervical lymph nodes	ERMS + UNB	N	Chemotherapy + radiotherapy	Disease-free survival
7	Majhi U (2012) ^[9]^	40	M	Superior nasal meatus + right cervical lymph nodes levels I–V	ERMS + PDNB	N	Chemotherapy + radiotherapy	Lost to follow-up
8	Kao WT (2015) ^[10]^	34	M	Beside the left scrotal testicle	PRMS + GN	N	Surgery + chemotherapy	Acute episode survival of pulmonary metastasis
9	Mahajan S (2019) ^[11]^	54	M	Left frontotemporal parietal lobe and dura mater	ERMS + GC	N	Surgery + chemotherapy	No recurrence at 7 mo postoperative
10	Davidson B (2021) ^[12]^	72	F	Uterus	SCR + GC	DICER 1, TP 53, PTEN Missense mutation	Surgery + chemotherapy	With metastatic lesions and survival
11	This case	23	M	Right frontotemporal region	ERMS + PDNB	NOTCH2 Frameshift mutation (meaning unclear)	Surgery + chemotherapy	Death due to illness within 1 yr

ARMS = alveolar rhabdomyosarcoma, ERMS = embryonal rhabdomyosarcoma, GC = ganglion cell, GN = ganglioneuroma, GNB = ganglioneuroblastoma, MEM = malignant ectomesenchymoma, PDNB = poorly differentiated rhabdomyosarcoma, PRMS = pleomorphic rhabdomyosarcoma, UNB = undifferentiated neuroblastoma.

### 4.2. Imaging characteristics

Currently, imaging data are only available for 7 adult MEM cases, with computed tomography and magnetic resonance imaging being the most commonly used examination methods, primarily aimed at determining the tumor boundaries and extent of involvement. Computed tomography typically manifests as a soft tissue mass shadow with well- or ill-defined borders, demonstrating heterogeneous enhancement upon contrast scanning; magnetic resonance imaging typically shows uniformly enhancing lesions, with T1WI signals mostly isointense or slightly hypointense, and T2WI signals slightly hyperintense or isointense, often accompanied by significant peritumoral edema.

### 4.3. Pathological characteristics

#### 4.3.1. Histological characteristics

The histological morphology of MEM can be summarized as “two components, multiple configurations”: The tumor tissue is distributed in nests or small nodules without a capsule, with fibrous septa observed in some areas, and extensive infiltration into surrounding soft tissues visible in most cases. The 2 components of the tumor refer to the mesenchymal and neuroectodermal components. The mesenchymal components are all RMSs, similar to juvenile cases, and various RMS subtypes can occur, including embryonal rhabdomyosarcoma, alveolar rhabdomyosarcoma (ARMS), spindle cell rhabdomyosarcoma (SRMS), and pleomorphic rhabdomyosarcoma, among others. ERMS is the most common subtype. In contrast to the neuroectodermal components in juvenile cases, the pathological types were morphologically more primitive and higher-risk tumors, primarily (6 cases) undifferentiated or poorly differentiated neuroblastomas (NBs), followed by only 2 cases containing pure ganglion cells, 2 cases of ganglioneuroma, and one case of ganglioneuroblastoma. Currently, there are no reported cases of adult patients with primitive neuroectodermal tumor, schwannoma, or a small number of other mesodermal mesenchymal components. The subtypes of the 2 components determine the diverse histological patterns of the tumor. Both ERMS and NB exhibit small round or oval cells; ARMS exhibits alveolar-like structures; SRMS is predominantly composed of spindle cells; and pleomorphic rhabdomyosarcoma shows large pleomorphic cells with varying morphologies. The tumor contains varying numbers of rhabdomyoblast-like cells and ganglion-like cells, while the NB component also exhibits pseudorosette structures, epithelioid acini, and rosette formations.^[11]^ The 2 components are mixed or interwoven, either alternately distributed or transitioning into each other. Their proportions may be similar, or one component may dominate. The stroma may exhibit nonspecific changes such as mucinous degeneration, fibrous deposition, or extravasation of red blood cells. It can be difficult to distinguish between the 2 components based solely on microscopic morphology, especially in ERMS cases admixed with undifferentiated or poorly differentiated NB and needle biopsy cases where misdiagnosis or missed diagnosis is highly likely. Indeed, both case 3 and the cases from our hospital were initially misdiagnosed.

#### 4.3.2. Immunophenotypic and molecular pathological characteristics

The most classic immunophenotype is the respective expression of corresponding markers by both components. The RMS component expresses muscle-specific actin, desmin, MyoD1, and Myogenin, while the neuroectodermal component expresses NSE, Syn, CgA, and S-100 protein. When interpreting immunohistochemical results, attention should be paid to excluding the co-expression of myogenic and neurogenic markers in the same cell type. With the continuous development of molecular biology, some studies suggest that MEM may exhibit genetic overlap with RMS. In juvenile cases, reports indicate that MEM, similar to ERMS, exhibits trisomy of chromosomes 2, 8, and 11.^[13]^ MEM containing ERMS components exhibits high-frequency *HRAS* mutations consistent with ERMS,^[14]^ while MEM with ARMS components demonstrates corresponding abnormal fusions such as *PAX3-FOXO1/PAX7-FOXO1*.^[15]^ Currently, there have been no reports detecting *MYOD1* mutations in MEM containing SRMS components (including the case from our hospital). In a previous study, molecular array analysis of 51 pediatric tumors revealed overlaps between malignant intracranial MEM and malignant peripheral nerve sheath tumors.^[16]^ Recently, MEM with *BCOR-CCNB3* gene fusion has also been detected.^[17]^ However, among adult cases, only 2 have undergone relevant sequencing analysis to date. In the cases located in the uterus and pelvic region, missense mutations were identified in *DICER1, TP53*, and *PTEN*, which resemble some RMS cases frequently occurring in the genital area. However, no significant specific molecular alterations were detected in the case from our hospital. Based on a comprehensive analysis of relevant literature and cases from our hospital, we preliminarily infer that the MEM phenotype may represent a heterogeneous tumor group with diverse genotypes and origins. Primitive neural crest cells encounter various gene regulations during differentiation, with some cells being relatively prone to specific genetic alterations that lead to differentiation toward RMS. Moreover, the predisposition to genetic changes may vary across different anatomical sites. Intracranial tumors are prone to complex changes similar to malignant peripheral nerve sheath tumors, while urogenital sites are more likely to develop *DICER1* alteration-related MEM, and other sites exhibit changes more closely resembling ERMS and ARMS. As the current number of cases is limited, the above inferences require further research and verification. In summary, the key to pathological diagnosis of MEM lies in the identification of morphology and immunophenotype. For adult cases with relatively primitive cytomorphology shown under the microscope, especially cases involving the head and neck region or needle biopsy, unique attention should be paid to the possibility of MEM during differential diagnosis.

### 4.4. Differential diagnosis

Adult MEMs primarily require differentiation from several types of lesions. Tumors comprising only one of these components, including RMS and neuroectodermal tumors, which are the foremost differential diagnoses for MEM. In some cases, distinguishing between these tumors based solely on morphology is challenging, and immunohistochemical markers may show some overlap. However, a comprehensive differential diagnostic approach and accurate interpretation of immunohistochemical results serve as crucial points for differential diagnosis; Other single-type soft tissue tumors accompanied by certain myogenic or neurogenic differentiation: primarily malignant melanoma, BCOR-CCNB3 fusion sarcoma, and osteosarcoma, among others. The key to differential diagnosis also lies in the interpretation of immunohistochemical results. MEM must exhibit separate expression of their corresponding markers in both components, while malignant melanoma widely expresses melanocytic markers such as HMB45. Osteosarcoma may show tumorous bone formation, among other features; and Mixed tumors with overlapping morphologies and similar phenotypes, mainly including Mesenchymal chondrosarcoma, teratomas, malignant triton tumors, and neuromuscular vagal hamartoma: Mesenchymal chondrosarcoma demonstrates negative expression of myogenic markers and molecular detection shows *HYE1-NCOA2* fusion gene. The other 3 may contain myoblasts and/or neuroectodermal components. However, teratomas consist of multiple germ layer components, whereas the latter 2 do not exhibit malignant rhabdomyoblastic differentiation.

### 4.5. Treatment and prognosis

The clinical behavior of MEM resembles that of RMS, and a treatment approach similar to that for RMS is recommended. Juvenile cases treated with multimodal therapy, including surgery, chemotherapy, and radiotherapy, generally achieve favorable prognoses. We summarized adult cases and found that patient prognosis is related to whether the tumor can be completely resected and the differentiation degree of the 2 components. For tumors that can be completely resected, with RMS components being ERMS, ARMS, or SRMS, and neuroectodermal components being well-differentiated tumors such as ganglion cell or ganglioneuroma, a treatment plan combining surgery with chemotherapy is adopted. Even in cases where local lymph node metastasis has occurred, patients still show favorable prognosis, with no recurrence or death observed during follow-up periods ranging from 7 months to 6 years post-treatment. For cases where the RMS component is an intermediate variant and the neuroectodermal component is undifferentiated or poorly differentiated NB, the combination of chemotherapy and radiotherapy can effectively improve treatment outcomes and prognosis. Genetic testing can identify potential therapeutic targets, such as the discovery of uterine cases with co-mutations in *DICER1* and *PTEN*. The serine/threonine-protein kinase (AKT) inhibitor Capivasertib, which targets *PTEN*, has now been approved for clinical use, offering the potential for precision therapy to bring greater benefits to patients by delaying or avoiding the need for radiotherapy and chemotherapy.

## 5. Conclusion

Adult MEM is a biphenotypic sarcoma composed of rhabdomyosarcoma and neuroectodermal tissues. Morphological and immunohistochemical interpretation is crucial for diagnosis. treatment approaches and prognosis are correlated with the completeness of tumor resection and the differentiation degree of both components.

## Acknowledgments

We thank LetPub (www.letpub.com.cn) for its linguistic assistance during the preparation of this manuscript.

## Author contributions

**Conceptualization:** Le Xie, Rongjun Mao.

**Data curation:** Le Xie, Yingxin Huang, Rongjun Mao.

**Formal analysis:** Le Xie, Yingxin Huang.

**Investigation:** Le Xie, Yingxin Huang.

**Writing – original draft:** Le Xie, Yingxin Huang.

**Writing – review & editing:** Le Xie, Rongjun Mao.
